# Phytochemicals from Bark Extracts and Their Applicability in the Synthesis of Thermosetting Polymers: An Overview

**DOI:** 10.3390/ma17092123

**Published:** 2024-04-30

**Authors:** Tomasz Szmechtyk, Magdalena Małecka

**Affiliations:** Department of Physical Chemistry, Faculty of Chemistry, University of Łódź, Pomorska 163/165, 90-236 Łódź, Poland; magdalena.malecka@chemia.uni.lodz.pl

**Keywords:** phytochemicals, thermosetting polymers, bark extraction, TPC, sustainable polymers

## Abstract

This review focuses on recent research on the phytochemicals found in bark from different trees and their potential to be used as substrates for the synthesis of thermosetting resins. Recent studies about the influence of each bark harvesting step on the extracted phytochemicals, from debarking to extraction, are investigated. A comparison of bark extracts in terms of the correlation between extraction conditions and efficiency (based on the total phenolic content (TPC) and extraction yield) is presented for six groups of trees (Norway spruce, pine species, other conifers, oak species, other deciduous trees of the north temperate zone, tropical and subtropical trees) and evaluated. The evaluation revealed that there is an interesting relationship between the extraction time and the type of solvent for some types of tree bark. It was found that a relatively short extraction time and a solvent temperature close to the boiling point are favourable. The latest research on the application of bark extracts in different types of thermosetting resins is described. This review discusses the attractiveness of bark extracts in terms of functional groups and the possibilities arising from extractable phytochemicals. In addition, different approaches (selective versus holistic) and methods of application are presented and compared.

## 1. Introduction

Petroleum-based compounds are still the main source of reagents for polymer synthesis. However, current research is increasingly focusing on the development of alternative bio-based materials. They are especially relevant for thermosetting resins, which cannot be widely recycled like thermoplastics. These activities result from both ecological and economic issues, and therefore, the management of waste of natural origin is of particular importance as a double benefit. A potential supplier of this type of bio-waste could be the wood industry, whose annual global production is 5 billion m^3^ of wood products [1]. The primary by-product is bark, which is not included in this annual production, as it is mainly used as fuel in sawmills. Recently, the use of bark in horticulture has increased the market share of bark, but its use is still relatively low (about 30% and 15% of hardwood and softwood bark supplies in the USA, respectively) [2].

The share of bark in the total volume of a tree or the total weight of a tree depends on the species, size and age of the tree and on the measurement and calculation methods [3,4]. Also, regarding the difference between inner and outer bark, the density of the bark is lower (in the case of inner bark) or much lower (in the case of outer bark) than the density of the wood [5]. Nevertheless, it can be assumed that the volume fraction of bark varies between 10% and 25% for most tree species at the age appropriate for felling. This gives the global amount of bio-waste ranging from 0.56 to 1.67 billion m^3^. Furthermore, the approximate quantitative determination of extractable compounds from bark is difficult due to the age and species of the tree, the maturity of the bark [6], the season of bark harvesting, soil conditions and the extraction method (the type of solvent is especially crucial) [6,7,8]. Rough estimations can be made with bark densities in the range of 200–700 kg/m^3^ and extraction yields in the range of 5–35% [6,7,9,10,11,12]. It can be assumed that the amount of extractable compounds from bark that is obtained annually by the wood industry is about 80 million metric tons (for values averaged over three ranges). This value corresponds to 20% of the world’s plastic production in 2021 (390.7 million metric tons) and almost double the global demand for thermosetting resins in 2021 (49.2 million metric tons) [13].

**Figure 1:**
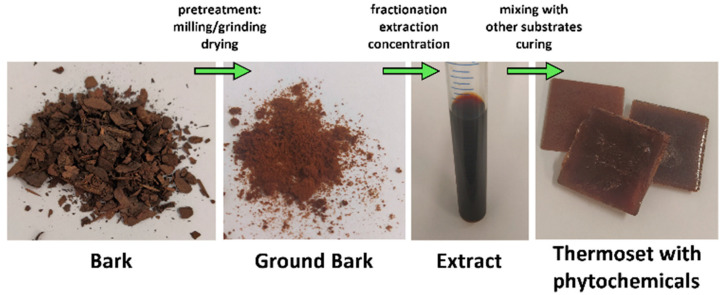
Simplified sourcing process of transforming bark into thermosetting resin.

The aim of this review was to investigate the sourcing process of phytochemicals from bark as extracts and their applicability to thermosetting resins (Figure 1). We present the collected data as an introduction to a discussion about the advantages and disadvantages of phytochemicals and the ways they are applied in thermosets.

## 2. Bark Harvesting

### 2.1. Types of Bark

Most species of trees have persistent bark that should only be harvested industrially as a by-product of felling. It is important to avoid causing unnecessary damage to the bark because this can result in an increased risk of tree disease and pest infestation. Strategies for obtaining persistent bark are therefore limited by the felling time. Generally, it is important to consider circumstances such as the season of felling, weather conditions, or the place of growth to better understand their impact on bark phytochemistry. An exception to the aforementioned rule is cork oak (*Quercus suber*), which has highly valuable bark (cork), resistant heartwood and the ability to regrow new outer bark. Cork is harvested industrially from living trees, but the process is very complex, and trees are still exposed to harmful environmental conditions after harvesting [14].

**Figure 2:**
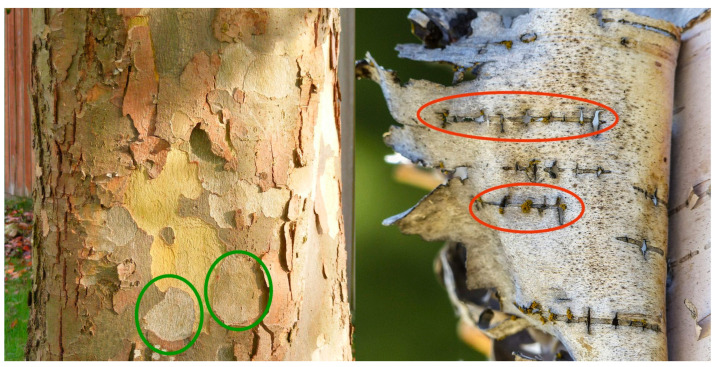
Two representatives of trees with exfoliating bark. The plane tree (**left**) has irregularly shaped flakes of bark (marked with green circles), which can be easily removed. Birch (**right**) has a thin paper-like bark with horizontal lenticels (marked with red circles), which are more fibrous.

Another group with different bark harvesting capabilities includes trees with exfoliating bark (Figure 2). The most notable representatives of this group are paperbark maple (*Acer griseum*), red maple (*A. rubrum*), most species of birch (*Betula papyrifera*, *B. pendula*, *B. maximowicziana*, *B. utilis*, *B. pubescens*, *B. nigra*, *B. alleghaniensis*, etc.), all eight species of plane tree (*Platanus*) [15], Chinese elm (*Ulmus parvifolia*), lacebark pine (*Pinus bungeana*), Sitka spruce (*Picea sitchensis*), various species of juniper (*Juniperus communis*, *J. virginiana* and *J. oxycedrus*), European yew (*Taxus baccata*) and shagbark hickory (*Carya ovata*). Exfoliation is a natural process and facilitates the harmless harvesting of the outer bark, but the bark should be carefully peeled in the early part of the growing season to avoid damaging the tree. Either way, the industrial harvesting of this type of bark from living trees is likely to be the subject of an environmental debate.

### 2.2. Debarking Methods

Bark that is harvested industrially during felling can be obtained using the dry or wet debarking process. The dry debarking (rotor debarking) method, used in the mechanical wood industry, only needs water if the logs are frozen [16]. The wet debarking (drum debarking) process, designed for the chemical wood industry, causes the release of water extractives with debarking effluent [17]. Consequently, the dry process provides the bark with higher amounts of phytochemicals, theoretically making it a more advantageous option. On the other hand, the liquid waste obtained after the wet process can still be a source of selected phytochemicals. Kemppainen et al. [18] performed extraction with hot tap water and proved that wet debarking can remove both carbohydrates and tannins from Norway spruce bark (*Picea abies*). Peeters et al. [19] also confirmed the similarity of the polyphenolic compositions of the water from the wet debarking process and bark press water to the compounds extracted (water as solvent) from Norway spruce bark. Bark extracts have a higher content of polyphenols than both types of wastewater. They found that bark press water, in particular, could still be a valuable source of phytochemicals with relatively low molecular weights. Multia [20] investigated the presence of stilbene glucosides in spruce bark and bark press water, the low levels of which suggest that they were largely extracted at an earlier stage (possibly during the wet debarking process). Neither of the above-mentioned wet processes is optimised as a source of phytochemicals, and due to this, common contaminants such as chlorides or metals [21] should be taken into account.

### 2.3. Seasonal Variations in Phytochemicals

The season of bark harvesting is another crucial factor. The examination of peach (*Prunus persica*) bark [22] indicates a marked correlation between the summer season and a high concentration of rutin (from May to August) and diacetylated *p*-coumaroylsucrose (from June to August). An inverse relationship was observed for catechin, persicoside, hesperetin-5-O-glucoside and 4′-O-methyltaxifolin-5-O-glucoside (high concentrations from September to April). Medic et al. [23] found increased contents of hydrojuglone glycosides (glucoside, rhamnoside, pentoside) and naphthoquinones in walnut *(Juglans regia*) bark extracts from the summer season (June–September). Most of the water-extractable phytochemicals from the bark of willow (*Salix* sp.) have higher concentrations in autumn and winter (triandrin, sucrose, raffinose, (+)-catechin, salicin), with the exception of glucose and fructose, whose contents are highest in July and relatively high throughout the year (lowest in September) [24]. The total phenolic content (TPC) and catechin content of water–ethanol (50% and 70% EtOH) extracts from the winter and spring bark of three *Acacia* species (*A. farnesiana*, *A. longifolia*, *A. tortilis*) were compared by Gabr et al. [25]. The only visible patterns were for the TPC of *A. tortilis* (significantly higher values for winter bark extracts) and the catechin content of *A. farnesiana* (higher values for spring bark extracts). The examination of Ashoka tree (*Saraca asoca*) bark showed increased epicatechin content in the winter season (Hemant and Shishir in the Hindu calendar) and increased gallic acid content in the monsoon season (Varsha) and early winter (Hemant) [26]. The complex study of stilbenes and carbohydrates in Norway spruce (*P. abies*) bark indicates higher contents of stilbenes and free sugars in samples collected in winter and total non-structural carbohydrates (mainly due to the presence of starch) in samples collected at the turn of spring and summer [27,28]. Furthermore, the tannin yield is higher in winter spruce bark extracts [29]. Halmemies et al. [30] observed a similar trend of higher values of total phenolic content (TPC), monosaccharides and stilbenes for winter spruce bark extracts, while the levels of organic acids, alcohols, flavonoids and distilbenes were similar for extracts in both seasons. Although recent studies of spruce bark provide relatively comprehensive information on extracts from different seasons, most studies of seasonal variability lack information about all relevant external factors (weather conditions, type of soil, pest activity). These factors, in addition to the different solvents, the variety of extraction procedures and the complex metabolism of trees, make predicting seasonal changes in the composition of phytochemicals challenging.

### 2.4. Storage of Bark

Storage is also an important factor that affects the phytochemical content. The contents of the bark extractives of Scots pine (*Pinus sylvestris*) [31] and Norway spruce (*P. abies*) [17] (soluble in acetone) decrease after 8 weeks of storage (both as piles and outdoors, from August to October). Spruce bark is also more susceptible to the loss of water extractives during storage in summer than in winter, which is the result of higher UV radiation and higher temperatures [30]. The activity of insects and microorganisms is also a factor accelerating degradation [29], which can be observed as the self-heating of biomass piles [31].

### 2.5. Pest Infestations and Fungal Infections

The phytochemistry of living trees is affected by insects and microorganisms. Zhao et al. reported a significant loss of stilbene glucoside, lignan and flavonoid contents in the inner bark of Norway spruce (*P. abies*) inoculated with five types of blue-stain fungi, which are associated with bark beetles [32]. On the other hand, these trees increase the production of terpenoid oleoresins and polyphenols in response. The main end products are catechin, catechin–epicatechin dimers, and taxifolin and its glucoside, which are toxic to bark beetles and their fungal associates [33]. Also, American beech (*Fagus grandifolia*) increases catechin content upon *Neonecteria* sp. infection [34]. These significant changes in the composition of the extract may be of interest because infested trees are often felled to stop the spread of beetles, and their bark is undesirable waste.

## 3. Extract Preparation and Analysis

### 3.1. Pre-treatment

Bark is often obtained in the form of relatively large flakes or strips, requiring pre-treatment such as milling or grinding. Samples can be fresh, freeze-dried or air-dried prior to fragmentation, with the advantage of freeze-drying being its lower impact on the phytochemical content compared to air-drying. The ground bark is often fractionated to separate the finer particles, which provide more efficient extraction (better solvent penetration) [35]. Additionally, the selection of bark compartments (inner bark, outer bark) may be crucial for the production of desirable chemicals [5].

### 3.2. Solvent Selection

The solvents most frequently chosen for bark extraction are water and simple alcohols (methanol and ethanol). They are relatively inexpensive and provide a satisfactory yield of phytochemicals. Mixtures of these solvents are prepared in various proportions, as they are often more effective than either one alone [11,36,37,38]. Other less popular solvents are 1,4-dioxan, acetone, dimethylformamide, diethyl ether, ethyl acetate, chloroform, hexane, benzene, isopropanol and acetonitrile [37,39]. More complex mixtures, such as deep eutectic solvents based on choline chloride, are also being tested [35,40], but these mixtures have so far been more effective as lignin solvents [41]. Although the selection of the solvent is crucial, higher yields of polyphenols and other phytochemicals can be obtained with the optimised parameters (especially time and temperature) of the applied extraction method. Also, the stability of extractables (especially polyphenols) should be taken into account for more demanding conditions. 

### 3.3. Extraction Method

Various methods for efficient extraction are widely applied. Simple extraction by maceration (ME) and conventional extraction (CE) with heating, stirring or shaking is still popular: 6.5% (5/77) and 28.6% (22/77) of all reported extractions for each bark–method combination (Table 1, Table 2, Table 3, Table 4, Table 5 and Table 6), respectively. With another 5 (6.5%) extractions without a specified method (CE/ME) [42], simple methods account for 41.6% of all reported extractions (32/77). More advanced but still relatively simple and inexpensive methods are Soxhlet extraction (SoxE, 5/77, 6.5%), ultrasound-assisted extraction (UAE, 27/77, 35.1%) and microwave-assisted extraction (MAE, 8/77, 10.4%). These three moderate methods account for 51.6% (39/77) of the extractions reported (mixed MAE-UAE method in one study [43] counted as one for this summary). Supercritical fluid extraction (SFE, 2/77, 2.6%, one mixed SFE-UAE method [44]), pressurised liquid extraction (PLE, 1/77, 1.3%) and accelerated solvent extraction (ASE, 4/77, 5.2%) are less popular (7/77, 9.1% in total). The effectiveness of all methods is restricted by the type of solvents and the set of parameters (again, time and temperature, as well as others specific to each method: pressure, frequency, power of microwaves, etc.).

### 3.4. Quantitative and Qualitative Analysis of Extracts

The qualitative and quantitative evaluation of extracted phytochemicals provides comprehensive information on their potential applications. The most common quantitative analysis is total phenolic content (TPC, expressed as milligrams of gallic acid per gram of dry weight of bark), which uses Folin–Ciocâlteu reagent to measure UV-Vis absorbance [45]. Another is the extraction yield, which is the percentage value of the extractive weight to the dry weight of extracted bark. Both were selected as key values in all the comparisons presented in the tables. Other popular quantitative methods are total flavonoid content (TFC) [36,42,43,44,46,47,48,49,50,51,52,53,54], total tannin content (TTC) [49,55,56,57] and antioxidant activity assays based on 2,2-diphenyl-1-picrylhydrazyl (DPPH) [36,37,38,40,42,43,44,47,48,49,50,51,52,53,54,55,56,57,58,59,60,61,62,63,64,65], 2,2’-azino-bis(3-ethylbenzothiazoline-6-sulfonic acid (ABTS) [36,37,38,43,46,47,48,53,55,56,57,58,62,64] and Ferric Reducing Antioxidant Power (FRAP) [36,37,38,42,43,44,46,47,48,53,58,61,64,66]. Antioxidant activity assays [11,60] with other assays and antimicrobial [36,38,44,55,56,57,63,67], inhibitory [38,53,55,57], cytotoxic [62,64] and antiplasmodial [53] activity measurement methods prove that the main areas of application of bark extractives are the medical, pharmaceutical and food industries. The only well-known quantitative method for the polymer industry is the estimation of the amount of reactive tannin towards formaldehyde, known as the Stiasny precipitation number [68,69]. The most common qualitative methods are liquid [6,36,37,38,42,44,57,58,61,62,63,67] and gas chromatography [42,44,63,64]), often combined with mass spectrometry (LC-MS, GC-MS) or with a photodiode array (LC-PDA). Less popular are phytochemical screening [52,59,60,64] and Fourier transform infrared spectroscopy (FTIR) [36,43,47], as they allow a more tentative identification of compounds or only the identification of classes of phytochemicals.

#### Chromatographic Identification of Phytochemicals: Conditions

The previously mentioned types of detectors—PDA and MS—can complement each other in liquid chromatography (as PDA-MS) [6,37,38,58,62], but a PDA alone was a slightly more common option here [36,42,44,57,61,63,67]. Two types of LC were predominantly used: ultra-pressure liquid chromatography (UPLC) and high-pressure liquid chromatography (HPLC). This was also reflected in the dimensions of the columns used. Most columns for UPLC were shorter. They were 100 mm long and had an internal diameter of 2.1 mm [36,62], 150 mm long with the same diameter [37] or 150 mm long with an internal diameter of 3 mm [6] or 4.6 mm [57,67]. HPLC columns were usually longer and had larger diameters, but this was not a rule. The standard dimensions of the normal-phase columns for HPLC were 250 mm × 4.6 mm [42,44,58]. The exceptions were the reverse-phase columns [38,61,63]. Anyway, all selected columns were C18. The separation temperature range was 25–35 °C. The flow rate ranged from 0.2 mL/min to 1.0 mL/min and the volume of injections from 5 µL to 20 µL. Methanol, acetonitrile, water and their solutions (concentrations from 0.1% to 2%) of acids (formic, acetic, trifluoroacetic and orthophosphoric) were chosen as mobile phases.

Gas chromatography was combined only with MS, and columns with the dimensions 30 m × 0.25 mm were used for all analyses. The heating procedures were different: from 40 °C to 260 °C (heating rate 4 °C/min, 10 min steps at 240 °C and 260 °C) [42], from 70 °C to 290 °C (heating rate 10 °C/min) [44] or from 60 °C to 300 °C (different heating rates were applied during the process: 3 °C/min, 2 °C/min and 10 °C/min) [64].

## 4. A Survey of Selected Recent Studies

**Figure 3:**
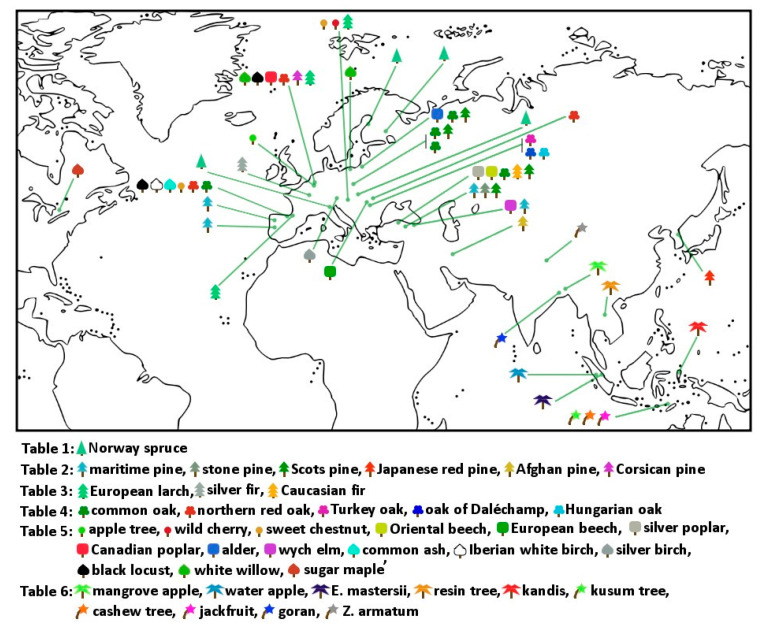
The locations of the tree species used to obtain all reported bark extracts. The sketch of the world map (author: OpenClipart-Vectors) was obtained as a free graphic from Pixabay GmbH (https://pixabay.com/vectors/map-world-geography-continents-117174/ (accessed on 15 January 2024)).

Recent studies (since 2018) on bark extracts are compared in tables, which are divided as follows: Table 1—Norway spruce (*Picea abies*); Table 2—different pine species (*Pinus* sp.); Table 3—other conifers; Table 4—different oak species (*Quercus* sp.); Table 5—other deciduous trees of the north temperate zone; and Table 6—trees of tropical and subtropical zones. The solvent type, the temperature of the process (if available, not applicable for MAE and ME), the extraction method (with additional information) and the time were selected as input parameters. The TPC, extraction yield and identified abundant compounds were chosen as the results for comparison. The evaluation of the presented data is informative due to the complex relationships between all input parameters (not only those presented hereafter but also those from sections “Bark Harvesting”, “Pre-treatment” and many others) and the results (extraction efficiency). Almost all TPC results were converted to milligrams of gallic acid equivalent per gram of dry weight of bark (mg GAE/g DWB) to avoid confusing comparisons between different forms of TPC expression. The two most popular forms were the aforementioned mg GAE/g DWB and milligrams of GAE per gram dry weight of extract (mg GAE/g DWE). The form mg GAE/g DWB was selected as a better source of information on the value of each extraction set (bark, solvent, extraction method) in terms of polyphenol yield. Another advantage of DWB as the denominator herein is the better comparability of extracts as products of bark because processes such as the concentration or fractionation of extracts make comparisons based on DWE less relevant. Conversions were made according to the equation DWB = DWE × Ey, where Ey is the extraction yield. The locations of the tree species used to obtain all reported bark extracts are shown in Figure 3. Most of the bark samples of these tree species were collected in the European region, with the exception of the species shown in Table 6, which are mainly from Southeast Asia. This trend is likely due to the European Green Deal and the sustainability initiatives that have resulted from it. For Asian countries, interest arises from the need to take advantage of the favourable climate and local flora.

### 4.1. Evaluation of Norway Spruce Bark Extracts (Table 1)

**Scheme 1:**
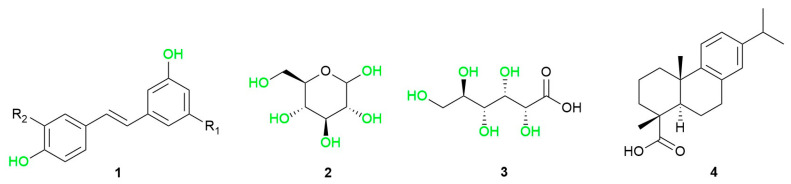
Structures of identified abundant compounds in bark of Norway spruce (*Picea abies*): (**1**) stilbenoids and stilbene glycosides (astringin: R_1_—beta-D-glucosyl, R_2_—OH; *trans*-resveratrol: R_1_—OH, R_2_—H; trans-isorhapontin: R_1_—β-D-glucosyl, R_2_—OCH_3_); (**2**) D-glucopyranose (glucose); (**3**) gluconic acid; (**4**) dehydroabietic acid.

Spinelli et al. [46] compared SFE and PLE with the most popular UAE for Norway spruce bark (Table 1), and UAE provided a competitive extraction yield with the highest total phenolic content (TPC) among them. Only PLE enables the production of comparable extracts with a higher total flavonoid content (TFC). A comparison of selected parameters for ASE [30,35] suggests that a shorter process time and water as a solvent (or a temperature closer to the boiling point of the solvent) may be favourable for higher TPC (for spruce bark). The highest TPC (110 mg GAE per g of dry weight of bark for bark obtained in winter) and extraction yield (35.7% for bark from summer) was obtained for ASE (1500 psi, 10 min static time) with high-purity water (at 120 °C) [30]. The compounds identified with relatively high yields were *trans*-resveratrol, dehydroabietic acid, glucose, gluconic acid, *trans*-isorhapontin and astringin (structures are presented in Scheme 1).

**Table 1:** Phytochemical extraction from Norway spruce (*Picea abies*) bark—comparison of selected recent studies (since 2018).

Ref.	Solvent Type, Temperature of Extraction	Extraction Method ^a^, Time	TPC (mg GAE /g DWB) ^b^	Extraction Yield (% DW)	Identified Abundant Compounds
[35]	ethanol 96.6%, 100 °C	ASE, 1500 psi, 20 min (steam exposure)	3.21	6.63	n/a
	ethanol 96.6%, 160 °C	ASE, 1500 psi, 30 min (steam exposure)	2.36	28.44	n/a
	ethanol 96.6%	MAE	3.21	n/a	n/a
	DES ^c^—choline chloride–malic acid 1:1 (*m/m*), 60 °C	CE (closed flask, continuous stirring), 1 h	9.00	14.68	n/a
	DES ^c^—choline chloride–maleic acid 1:1 (*m/m*), 60 °C		20.00	11.87	
	DES ^c^—choline chloride–glycerol 1:2 (m/m), 60 °C		17.00	11.40	
[46]	ethanol–water 10:90 (*v*/*v*), 40 °C	SFE, 100 bar, 105 min (dynamic time), 150 min (static time)	0.77 ± 0.02	2.86 ± 0.04	*trans*-resveratrol ^d^
	ethanol–water 20:80 (*v*/*v*), 40 °C		1.24 ± 0.07	3.07 ± 0.10	
	ethanol–water 40:60 (*v*/*v*), 40 °C		2.50 ± 0.03	3.12 ± 0.02	
	water, 160 °C	PLE, 50 bar, 5 min	33.45 ± 1.44	13.07 ± 0.86	
	ethanol, 180 °C		46.32 ± 2.17	12.79 ± 0.25	
	ethanol–water 70:30 (*v*/*v*), 54 °C	UAE (39 kHz, bath, 200 W), 60 min	54.97 ± 2.00	12.33 ± 0.58	
[17]	acetone, ~56 °C	SoxE, 15 min	n/a	11.83 ± 0.13 ^e^	n/a
[40]	DES13 ^c^, 60 °C	CE (closed flask, cont. stirring), 2 h	5.31 ± 0.04	n/a	n/a
	DES14 ^c^, 60 °C		5.96 ± 0.07	n/a	
[30]	ultra-high-quality water, 120 °C	ASE, 1500 psi, 10 min (static time)	111.0 (winter) ^e^ 89.4 (summer) ^e^	34.7 (winter) ^e^ 35.7 (summer) ^e^	dehydroabietic acid, glucose, gluconic acid, *trans*-isorhapontin, astringin

^a^ Types of extractions: SoxE—Soxhlet ex.; SFE—supercritical fluid ex.; PLE—pressurised liquid ex.; UAE—ultrasound-assisted ex.; CE—conventional ex.; ASE—accelerated solvent ex.; MAE—microwave-assisted ex. ^b^ TPC—total phenolic content, expressed as mg gallic acid equivalent per g dry weight of bark (mg GAE/g DWB); n/a—not available. ^c^ DES—deep eutectic solvent; DES 13 and DES 14—deep eutectic solvents based on choline chloride, lactic acid, 1,3-butanediol and water in molar ratios 1:4:1:1 and 1:5:1:1, respectively. All components were mixed to form a homogeneous liquid (60 °C; 30 min). Only the 2 best results are presented. ^d^ Trans-resveratrol was the only identified compound. ^e^ Extraction yield/TPC of fresh bark.

### 4.2. Evaluation of Pine Species Bark Extracts (Table 2)

**Scheme 2:**
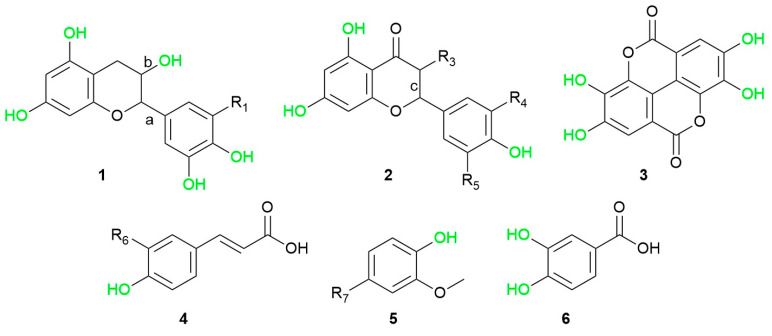
Structures of identified abundant compounds in bark of different pine species (*Pinus* sp.): (**1**) flavan-3-ols (catechin: R_1_—H, a, b—S, R (−)/R, S (+); epicatechin: R_1_—H, a, b—S, S (+)/R, R (−); gallocatechin: R_1_—OH, a, b—S, R (−)/R, S (+)); (**2**) other flavonoids (taxifolin: R_3_—OH, R_4_—H, R_5_—OH, c—single bond; myricetin: R_3_—OH, R_4_—OH, c—double bond, R_5_—OH; naringenin: R_3_—H, R_4_—H, R_5_—H, c—single bond; (**3**) ellagic acid; (**4**) caffeic acid: R_6_—OH; ferulic acid: R_6_—OCH_3_; (**5**) guaiacol: R_7_—H; 4-methylguaiacol: R_7_—CH_3_; 4-vinylguaiacol: R_7_—CH=CH_2_; (**6**) protocatechuic acid.

The highest TPC (163.6 mg GAE per g of dry bark) was obtained for maritime pine (*P. pinaster*) conventionally extracted (115 min) in an equal-volume ethanol–water mixture [36]. The highest extraction yields (17.5–18.5%) were obtained using simple methods (SoxE, CE/ME) for maritime pine (*P. pinaster*) and Scots pine (*P. sylvestris*) in alcohol–water mixtures [11,42]. Despite this, such comparisons between species of the *Pinus* genus have limited rationality (specific site of growth and growth conditions, slightly different phytochemistry). CE, the most popular extraction method for pine species, can be more accurately evaluated. The results for maritime pine (*P. pinaster*) [36,70] suggest that an extraction time of approximately 2 h and a temperature close to the boiling point (lower TPC for water at 82 °C) are optimal for this method and this type of bark. Equal or nearly equal volume ratios of water and ethanol are also optimal for higher TPC, but the results of Japanese red pine reveal the potential of acetonitrile and isopropanol as alternatives to ethanol [37]. All the abundant compounds mentioned in Table 2 are presented in Scheme 2.

**Table 2:** Phytochemical extraction from bark of different pine species (*Pinus* sp.) *—comparison of selected recent studies (since 2018).

Ref.	Solvent Type, Temperature of Extraction	Extraction Method ^a^, Time	TPC (mg GAE /g DWB) ^b^	Extraction Yield (% DW)	Identified Abundant Compounds
[11] mp	ethanol–water 50:50 (*v*/*v*), ~82 °C	SoxE, 4 h	73.48 ± 1.83	17.55 ± 0.16	n/a
	ethanol 96%, 78 °C		63.38 ± 1.26	17.08 ± 0.23	
	water, 100 °C		50.09 ± 4.70	ca. 8 ^c^	
[70] mp	water, 95 °C	CE (with ice on lid), 2 h	101.1 ± 4.0	7.5	n/a
[36] mp	water, 82 °C	CE, 115 min	48.1	n/a	gallocatechin, taxifolin, ellagic acid
	ethanol–water 30:70 (*v*/*v*), 82 °C		120.1	n/a	taxifolin, gallocatechin, naringenin, catechin, elagic acid
	ethanol–water 50:50 (*v*/*v*), 82 °C		163.6	n/a	taxifolin, naringenin, catechin, ellagic acid
	ethanol–water 70:30 (*v*/*v*), 82 °C		136.5	n/a	
	ethanol–water 90:10 (*v*/*v*), 82 °C		123.8	n/a	
[61] mp	water, ~100 °C	CE, 15 min	12.25 ± 0.03	n/a	catechin, taxifolin, protocatechuic acid
[61] sp	water, ~100 °C	CE, 15 min	14.77 ± 0.06	n/a	catechin, taxifolin, caffeic acid
[61] Sc	water, ~100 °C	CE, 15 min	5.42 ± 0.05	n/a	catechin, taxifolin, protocatechuic acid
[42] Sc	methanol–water 65:35 (*v*/*v*)	n/a (CE/ME)	88	18.33	myricetin, eleutheroside
[49] Sc	distilled water, 60 °C	CE (cont. stirring), 1 h	12.33 ± 1.48 7.25 ± 1.37 5.80 ± 1.24 4.38 ± 0.94 ^d^	n/a	n/a
[50] Sc	ethanol–water 60:40 (*v*/*v*), 50 °C	CE (cont. stirring), 20 min	ca 67 ^c^	–	catechin, epicatechin
[37] Jp	water, 60 °C	CE (extraction in heating mantle), 9 h	9.04	6.18	n/a
	ethanol–water 20:80 (*v*/*v*), 60 °C		22.19	8.34	
	ethanol–water 40:60 (*v*/*v*), 60 °C		24.33	9.52	
	ethanol–water 60:40 (*v*/*v*), 60 °C		20.15	11.76	
	ethanol–water 80:20 (*v*/*v*), 60 °C		14.76	10.21	
	ethanol, 60 °C		7.56	8.15	
	methanol–water 20:80 (*v*/*v*), 60 °C		16.76	8.36	
	methanol–water 40:60 (*v*/*v*), 60 °C		16.37	9.09	
	acetone–water 20:80 (*v*/*v*), 60 °C		20.26	8.41	
	acetone–water 40:60 (*v*/*v*), 60 °C		17.16	9.58	
	isopropanol–water 20:80 (*v*/*v*), 60 °C		24.51	10.11	
	isopropanol–water 40:60 (*v*/*v*), 60 °C		29,46	12.05	
	acetonitrile–water 20:80 (*v*/*v*), 60 °C		23.81	9.24	
	acetonitrile–water 40:60 (*v*/*v*), 60 °C		27.68	11.37	
[66] Ap	ethanol–water 70:30 (*v*/*v*), room temperature	ME, 72 h	560.65 ± 44.00 ^e^	n/a	catechin, ferulic acid, taxifolin, caffeic acid ^f^
[71] Cp	ethanol–water 50:50 (*v*/*v*), 50 °C	UAE, 1 h	27.9 ± 0.3	11.1	4-vinyl guaiacol, 4-methylguaiacol, guaiacol

* Compared pine species (abbreviations in the Ref. section): mp—maritime pine (*P. pinaster*); sp—stone pine (*P. pinea*); Sc—Scots pine (*P. sylvestris*); Jp—Japanese red pine (*P. densiflora*); Ap—Afghan pine (*P. eldarica*); Cp—Corsican pine (*Pinus nigra* subsp. *laricio*). ^a^ Types of extractions: SoxE—Soxhlet ex.; CE—conventional ex.; ME—ex. by maceration. ^b^ TPC—total phenolic content, expressed as mg gallic acid equivalent per g dry weight of bark (mg GAE/g DWB); n/a—not available. ^c^ Value obtained from an inaccurate graph. ^d^ In order from top to bottom: whole bark from the continental (cont.) zone, outer bark from the cont. zone, whole bark from the coastal (coa.) zone, outer bark from the coa. zone. ^e^ Incomparable, expressed as mg GAE per dry weight of extract (without extraction yield, TPC cannot be converted to mg GAE per dry weight of bark). ^f^ Quoted from their earlier article.

### 4.3. Evaluation of Other Conifers’ Bark Extracts (Table 3)

**Scheme 3:**
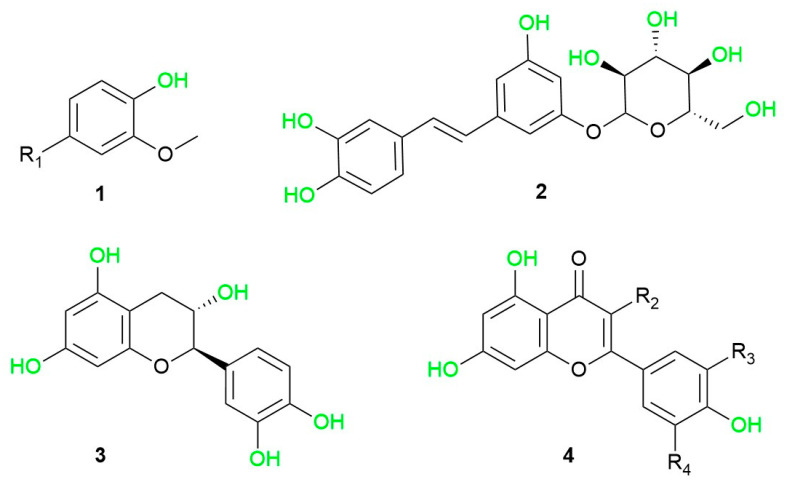
Structures of identified abundant compounds in bark of other conifers: (**1**) guaiacol: R_1_—H; 4-methylguaiacol: R_1_—CH_3_; 4-vinylguaiacol: R_1_—CH=CH_2_; (**2**) astringin; (**3**) (+)-catechin; (**4**) flavones (myricetin: R_2_—OH, R_3_—OH, R_4_—OH; isorhamnetin: R_2_—OH, R_3_—H, R_4_—OCH_3_; isorhamnetin glucoside: R_2_—β-D-glucosyl, R_3_—OH, R_4_—OCH_3_; quercetin glycoside: R_2_—β-D-glucosyl acetate, R_3_—H, R_4_—OH).

In this table, studies of European larch bark extracts (*Larix decidua*) and different firs (*Abies* sp. and *Cunninghamia lanceolata*) are presented together. Interestingly, the TPC results from larch studies are very diverse (from 6 to 145 mg GAE/g DWB) for relatively similar extraction conditions. However, temperature and time might be crucial parameters: all three extracts with low TPC were probably overheated (heating above 50 °C or exposure to microwaves for more than 1 h) [48]. The best TPC values were obtained by the UAE method (a horn sonicator provides a yield of polyphenols similar to that obtained by the ultrasound bath but 4 times faster). Polyphenols probably extracted from larch bark are temperature-sensitive, and longer exposure causes their decomposition and/or evaporation (the accompanying low extraction yields for these three extracts may also be indicative of this). Unfortunately, a qualitative analysis provides limited data: only astringin, 4-vinyl guaiacol, 4-methylguaiacol and guaiacol were identified (Scheme 3), but the last three are considered volatile. Only two studies on firs (*Abies* sp.) were collected, and the only possible conclusion is a relatively high extraction yield (around 20%) for alcohol–water mixtures as solvents. Compounds identified with relatively high yields were isorhamnetin glucoside, quercetin glycoside, isorhamnetin, (+)-catechin and myricetin (Scheme 3). The TPCs of Chinese fir (*Cunninghamia lanceolata*) bark extract indicate the better solubility of its polyphenols in ethanol (almost twice as high TPC compared to water extracts).

**Table 3:** Phytochemical extraction from bark of other conifers *—comparison of selected recent studies (since 2018).

Ref.	Solvent Type, Temperature of Extraction	Extraction Method ^a^, Time	TPC (mg GAE /g DWB) ^b^	Extraction Yield (% DW)	Identified Abundant Compounds
[58] El	ethanol–water 80:20 (*v*/*v*)	UAE (horn), 15 min	145.22 ± 6.11	n/a	astringin
[71] El	ethanol–water 50:50 (*v*/*v*), 50 °C	UAE, 1 h	143.7 ± 4	26.1	4-vinyl guaiacol, 4-methylguaiacol, guaiacol
[43] El	ethanol–water 50:50 (*v*/*v*)	MAE-UAE (simultaneous, power: 100–300 W), 30–120 s	90 ± 3	15.1 ± 0.1	n/a
[48] El	ethanol–water 50:50 (*v*/*v*), 58.26 °C	CE (with orbital shaker, speed 120 rpm), 94.27 min	0.83	7.73	n/a
	ethanol–water 50:50 (*v*/*v*), 65 °C	UAE (bath) 94.76 min	0.37	5.87	
	ethanol–water 50:50 (*v*/*v*)	MAE (power: 100 W), 62.66 min	0,88	8.21	
[6] sf	ethanol–water 50:50 (*v*/*v*)	ASE	n/a	21.63 ^c^	isorhamnetin glucoside, quercetin glycoside, isorhamnetin, (+)-catechin
[42] Cf	methanol–water, 65:35 (*v*/*v*)	n/a (CE/ME)	73	19.10	myricetin
[72] Ch	1% NaOH (aq), 90 °C	CE, 2 h	149.3	21.26 ± 0.81	n/a
	ethanol 95%	SoxE, (4–5 cycles/h), 7 h	285.6	5.04 ± 0.22	
	water, 90 °C	CE, 2 h	162.7	2.46 ± 0.14	

* Compared other conifers (abbreviations in the Ref. section): El—European larch (*Larix decidua*); sf—silver fir (*Abies alba*); Cf—Caucasian fir (*Abies nordmanniana*); Ch—Chinese fir (*Cunninghamia lanceolata*). ^a^ Types of extractions: UAE—ultrasound-assisted ex.; CE—conventional ex.; ASE—accelerated solvent ex.; MAE—microwave-assisted ex.; SoxE—Soxhlet ex. ^b^ TPC—total phenolic content, expressed as mg gallic acid equivalent per g dry weight of bark (mg GAE/g DWB); n/a—not available. ^c^ Average of mean values for all disc–tree configurations.

### 4.4. Evaluation of Oak Species Bark Extracts (Table 4)

**Scheme 4:**
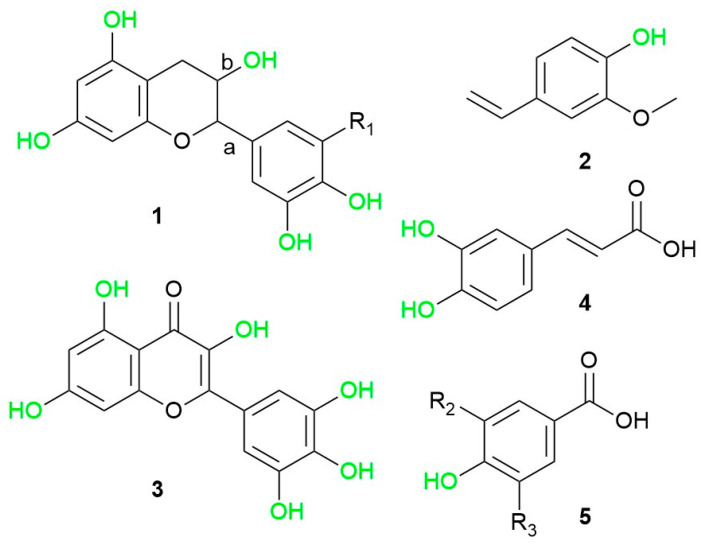
Structures of identified abundant compounds in bark of different oak species: (**1**) flavan-3-ols (catechin: R_1_—H, a, b—S, R (−)/R, S (+); gallocatechin: R_1_—OH, a, b—S, R (−)/R, S (+)); (**2**) 4-vinylguaiacol; (**3**) myricetin; (**4**) caffeic acid; (**5**) p-hydroxybenzoic acid: R_2_—H, R_3_ —H; vanillic acid: R_2_—OCH_3_, R_3_—H; gallic acid: R_2_—OH, R_3_—OH.

The highest TPC value (79.3 ± 0.8 mg GAE/g DWB) was obtained from the bark of common oak (*Quercus robur*) as a result of a short (20 min) CE (room temperature) in an ethanol–water mixture (60:40, *v*/*v*) [51]. The short process time may be crucial for a better extraction of polyphenols from this type of bark, because a similar experiment at an elevated temperature (50 °C) gave comparable results (approx. 77 mg GAE/g DWB) [50]. Furthermore, the TPC values for longer extraction times were significantly lower, which may indicate that the polyphenols obtained are sensitive even to long-term exposure to mild heating. A relatively high TPC value for UAE heated for a long time (1 h, 50 °C) [47] led us to assume that UAE may be a more effective technique if it is used for a shorter period of time. The extraction yields were mentioned in only four studies, and the highest one (18.75%) was obtained by an unspecified extraction (CE/ME) in a mixture of methanol with water (65:35, *v*/*v*). The bark of other oak species was extracted by assisted extraction (UAE or MAE), and the TPC values exceeded 200 mg GAE/g DWB, with two exceptions (both were heated for one hour) [47,71]. These results partially confirm the hypothesis mentioned above about the advantage of UAE over CE for common oak bark extraction. On the other hand, a complex comparison of TPC values obtained by MAE and UAE (shorter and longer process times for the two methods) reveals microwaves to be a well-suited assisting technique for the fast heating of most oak species bark, especially for ethanol–water mixtures as solvents (highest TPC values for all oak species were obtained by MAE, 650 W, 18 min). Catechin and gallic acid are compounds identified for at least two species of oak. Other identified and abundant compounds for single species were *p*-hydroxybenzoic acid, epicatechin, myricetin (common oak), 4-vinyl guaiacol (northern red oak), vanillic acid (oak of Daléchamp) and caffeic acid (Hungarian oak). Their structures are presented in Scheme 4.

**Table 4:** Phytochemical extraction from bark of different oak species (*Quercus* sp.) *—comparison of selected recent studies (since 2018).

Ref.	Solvent Type, Temperature of Extraction	Extraction Method ^a^, Time	TPC (mg GAE /g DWB) ^b^	Extraction Yield (% DW)	Identified Abundant Compounds
[50] co	ethanol–water 60:40 (*v*/*v*), 50 °C	CE (cont. stirring), 20 min	ca 77 ^c^	n/a	catechin, epicatechin, *p*-hydroxybenzoic acid
[49] co	distilled water, 60 °C	CE (cont. stirring), 1 h	18.09 ± 3.50 10.28 ± 2.05 17.68 ± 3.14 8.25 ± 1.55 ^d^	n/a	n/a
[51] co	water, rt	CE (cont. stirring), 20 min	60.4 ± 1.3	n/a	n/a
	ethanol–water 60:40 (*v*/*v*), rt		79.3 ± 0.8	n/a	
[42] co	methanol–water 65:35 (*v*/*v*)	n/a (CE/ME)	48	18.75	myricetin
[47] co	ethanol–water 50:50 (*v*/*v*), 50 °C	UAE, 1 h	61.25 ± 1.50	10.03 ± 0.31	n/a
[47] no	ethanol–water 50:50 (*v*/*v*), 50 °C	UAE, 1 h	8.84 ± 0.10	3.20 ± 0.07	n/a
[71] no	ethanol–water 50:50 (*v*/*v*), 50 °C	UAE, 1 h	91.9 ± 3.2	17.3	4-vinyl guaiacol
[55] no	water, 70 °C	UAE (40 kHz), 15 min	203.58 ± 3.25	n/a	n/a
	ethanol–water 50:50 (*v*/*v*), 70 °C	UAE (40 kHz), 15 min	226.79 ± 1.54	n/a	
	water	MAE (850 W), 30 min	216.47 ± 1.19	n/a	
	ethanol–water 70:30 (*v*/*v*)	MAE (650 W), 18 min	321.08 ± 3.23	n/a	
[56] To	water	MAE (850 W), 30 min	382.26 ± 0.97	n/a	n/a
	ethanol–water 70:30 (*v*/*v*)	MAE (650 W), 18 min	403.73 ± 7.35	n/a	
[57] oD	water	UAE (40 kHz), 15 min	ca 290 ^c^	n/a	gallic acid, catechin, vanillic acid
	ethanol–water 70:30 (*v*/*v*)	UAE (40 kHz), 15 min	ca 285 ^c^	n/a	
	water	MAE (850 W), 30 min	ca 315 ^c^	n/a	
	ethanol–water 70:30 (*v*/*v*)	MAE (650 W), 18 min	ca 370 ^c^	n/a	
[57] Ho	water	UAE (40 kHz), 15 min	ca 310 ^c^	n/a	caffeic acid, catechin, gallic acid
	ethanol–water 70:30 (*v*/*v*)	UAE (40 kHz), 15 min	ca 330 ^c^	n/a	
	water	MAE (850 W), 30 min	ca 350 ^c^	n/a	
	ethanol–water 70:30 (*v*/*v*)	MAE (650 W), 18 min	ca 355 ^c^	n/a	

* Compared oak species (abbreviations in Ref section): co—common oak (*Quercus robur*); no—northern red oak (*Quercus rubra*); To—Turkey oak (*Quercus cerris*); oD—oak of Daléchamp (*Quercus dalechampii*); Ho—Hungarian oak (*Quercus frainetto*). ^a^ Types of extractions: UAE—ultrasound-assisted ex.; CE—conventional ex.; MAE—microwave-assisted ex. ^b^ TPC—total phenolic content, expressed as mg gallic acid equivalent per g dry weight of bark (mg GAE/g DWB); n/a—not available. ^c^ Value obtained from an inaccurate graph. ^d^ In order from top to bottom: whole bark from the continental (cont.) zone, outer bark from the cont. zone, whole bark from the coastal (coa.) zone, outer bark from the coa. zone; rt—room temperature.

### 4.5. Evaluation of Bark Extracts from Other Deciduous Trees of North Temperate Zone (Table 5)

The highest comparable (expressed as mg GAE per gram of dry weight of bark) TPC value (174.25 ± 16.95) was obtained for the sweet chestnut bark extract obtained by UAE with a horn sonicator (15 min) [58]. The TPC of the sweet chestnut bark bath/UAE-heated extract was significantly lower (58.87 ± 2.24 mg GAE/g DWB), which may suggest that the temperature (at least 50 °C) is more harmful to these polyphenols than to European larch polyphenols.

**Scheme 5:**
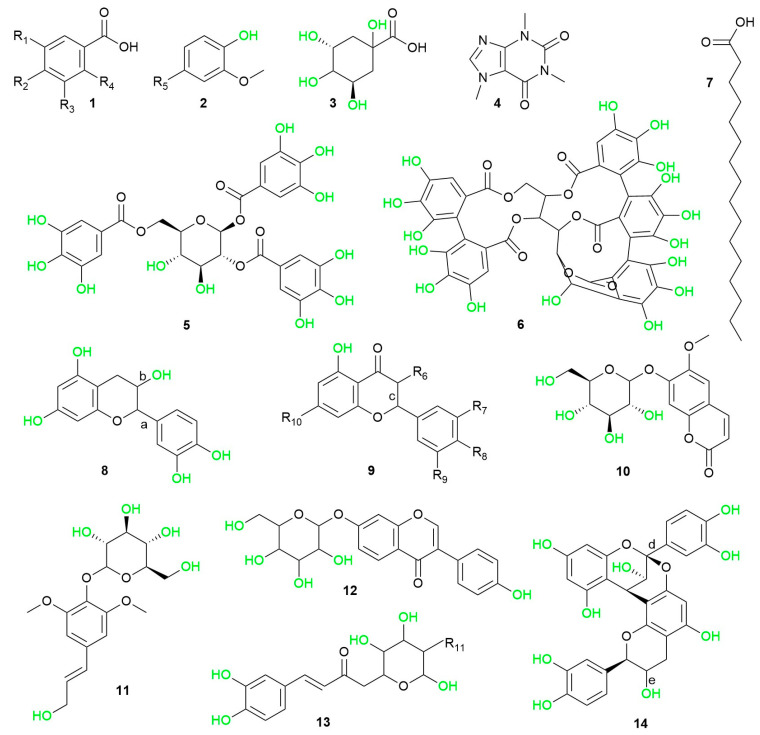
Structures of identified abundant compounds in bark of other deciduous trees of north temperate zone: (**1**) phenolic acids (benzoic acid: R_1_—H, R_2_—H, R_3_—H, R_4_—H; gallic acid: R_1_—OH, R_2_—OH, R_3_—OH, R_4_—H; vanillic acid: R_1_—OCH_3_, R_2_—OH, R_3_—H, R_4_—H; *p*-hydroxybenzoic acid: R_1_—H, R_2_—OH, R_3_—H, R_4_—H; phthalic acid: R_1_—H, R_2_—H, R_3_—H, R_4_—COOH); (**2**) guaiacol: R_5_—H; 4-methylguaiacol: R_5_—CH_3_; 4-vinylguaiacol: R_5_—CH=CH_2_; (**3**) quinic acid; (**4**) caffeine; (**5**) trigalloyl-HHDP-glucose; (**6**) vescalagin; (**7**) palmitic acid; (**8**) flavan-3-ols (catechin: a, b—S, R (−)/R, S (+); (-)-epicatechin: R_1_—H, a, b—R, R); (**9**) other flavonoids (myricetin: R_6_—OH, R_7_—OH, R_8_—OH, R_9_—OH, R_10_—OH, c—double bond; quercetin: R_6_—OH, R_7_—H, R_8_—OH, R_9_—OH, R_10_—OH, c—double bond; luteolin-O-hexoside: R_6_—H, R_7_–OH, R_8_—β-D-glucosyl, R_9_—H, R_10_—OH, c—double bond; apigenin-O-hexoside: R_6_—H, R_7_—H, R_8_—OH, R_9_—H, R_10_—β-D-glucosyl, c—double bond; taxifolin: R_6_—OH, R_7_—H, R_8_—OH, R_9_—OH, R_10_—OH, c—single bond; taxifolin-3-glucoside: R_6_–β-D-glucosyl (S), R_7_—H, R_8_—OH, R_9_—OH, R_10_—OH, c—single bond; taxifolin-7-glucoside: R_6_—OH (S), R_7_—H, R_8_—OH, R_9_—OH, R_10_—β-D-glucosyl (R), c—single bond; kaempferol-O-hexoside: R_6_—β-D-glucosyl, R_7_—H, R_8_—OH, R_9_—H, R_10_—OH, c—double bond; (**10**) scopolin; (**11**) syringin; (**12**) daidzein-O-hexoside; (**13**) caffeoyl hexose: R_11_—OH; caffeoyl hexose deoxyhexoside: R_11_—deoxyhexosyl; (**14**) procyanidin dimer type A (A1: e—S; A2: d—S, e—R).

A complex study of European beech extracts obtained by MAE was carried out, taking into account different microwave power values [38]. Water MAE beech extracts had the highest TPC values for the shortest extraction time (for all microwave power values). For equal-volume water–ethanol MAE beech extracts, a reverse trend was observed. The 80% ethanol MAE beech extracts were not clearly correlated with microwave power and time. Catechin and vanillic acid were identified in beech bark in two independent studies. All structures of abundant compounds mentioned in Table 5 (except unspecified eleutheroside) are presented in Scheme 5.

**Table 5:** Phytochemical extraction from bark of other deciduous trees of north temperate zone *—comparison of selected recent studies (since 2018).

Ref.	Solvent Type, Temperature of Extraction	Extraction Method ^a^, Time	TPC (mg GAE /g DWB) ^b^	Extraction Yield (% DW)	Identified Abundant Compounds
[44] at	subcritical water, 150 °C	SFE-UAE ^c^ (40 bar, 3 Hz), 40 min	31.47 ± 1.86	n/a	gallic acid, catechin, benzoic acid, guaiacol
[58] wc	ethanol–water 80:20 (*v*/*v*)	UAE (horn), 15 min	112.88 ± 17.27	n/a	-O-hexosides of: (luteolin, apigenin, daidzein, taxifolin, kaempferol), scopolin
[58] sc	ethanol–water 80:20 (*v*/*v*)	UAE (horn), 15 min	174.25 ± 16.95	n/a	trigalloyl-HHDP-glucose, quinic acid, vescalagin
[47] sc	ethanol–water 50:50 (*v*/*v*), 50 °C	UAE, 1 h	58.87 ± 2.24	9.27 ± 0.18	n/a
[42] Ob	methanol–water 65:35 (*v*/*v*)	– (CE/ME)	42.04	15.50	n/a
[67] Eb	distilled water, 85–90 °C	CE (water bath with shaking), 45 min	22.95 ± 0.07	n/a	catechin, vanillic acid, taxifolin, syringin
[38] Eb	water	MAE (300 ^c^, 450 ^d^, 600 ^e^, 800 ^f^ W), 2, 3, 4 min	47.44–51.53 ^c^ 48.19–56.79 ^d^ 52.79–59.10 ^e^ 55.68–72.31 ^f^	n/a	catechin, vanillic acid, (-)-epicatechin
	ethanol–water 50:50 (*v*/*v*)		67.27–76.46 ^c^ 66.43–77.53 ^d^ 71.91–72.43 ^e^ 72.46–73.32 ^f^	n/a	catechin, (-)-epicatechin, vanillic acid
	ethanol–water 80:20 (*v*/*v*)		61.87–64.77 ^c^ 66.00–67.86 ^d^ 69.81–70.95 ^e^ 64.12–66.07 ^f^	n/a	catechin, (-)-epicatechin, vanillic acid
[42] sp	methanol–water 65:35 (*v*/*v*)	–(CE/ME)	100	19.43	myricetin, eleutheroside, quercetin
[71] Cp	ethanol–water 50:50 (*v*/*v*), 50 °C	UAE, 1 h	56 ± 0.4	16.8	4-vinyl guaiacol, 4-methylguaiacol
[49] al	distilled water, 60 °C	CE (continuous stirring), 1 h	29.00 ± 5.33 13.42 ± 1.41 12.18 ± 1.53 4.92 ± 0.51 ^g^	n/a	n/a
[70] we	water, 95 °C	CE (with ice on lid), 2 h	407.05 ^h^	5.18	n/a
[47] ca	ethanol–water 50:50 (*v*/*v*), 50 °C	UAE, 1 h	49.91 ± 1.63	15.77 ± 0.14	n/a
[47] Ib	ethanol–water 50:50 (*v*/*v*), 50 °C	UAE, 1 h	21.99 ± 0.15	5.09 ± 0.06	n/a
[73] sb	methanol, 50 °C	UAE (bath, 35 kHz), 3 h	79.43	17.74 ± 1.64	n/a
[47] bl	ethanol–water 50:50 (*v*/*v*), 50 °C	UAE, 1 h	5.49 ± 0.18	3.08 ± 0.18	n/a
[71] bl	ethanol–water 50:50 (*v*/*v*), 50 °C	UAE, 1 h	25.3 ± 0.3	9.5	4-vinyl guaiacol
[71] ww	ethanol–water 50:50 (*v*/*v*), 50 °C	UAE, 1 h	102.6 ± 4.2	23.8	4-vinyl guaiacol, phenol
[62] ww	methanol–water 70:30 (*v*/*v*), 40 °C	UAE (with chloroformpre-extraction), 30 min	23.30 ± 0.17 ^k^	34.6	caffeoyl hexose, A-type procyanidin dimers, caffeoyl hexose-deoxyhexoside, (−)-epicatechin,
[63] sm	acetone, rt	CE (stirring), 6 h	190 ± 10 inner bark (IB) 292.67 ± 11.02 outer bark (OB) ^h^	6.06 ± 0.89 (IB) 7.82 ± 0.33 (OB)	IB: caffeine, *p*-hydroxybenzoic acid, palmitic acid OB: gallic acid, *p*-hydroxybenzoic acid, phthalic acid

* Compared deciduous trees of north temperate zone (abbreviations in Ref. section): at—apple tree (*Malus domestica*); wc—wild cherry (*Prunus avium*); sc—sweet chestnut (*Castanea sativa*); Ob—Oriental beech (*Fagus orientalis*); Eb—European beech (Fagus sylvatica); sp—silver poplar (*Populus alba*); Cp—Canadian poplar (*Populus × canadensis*); al—alder (*Alnus glutinosa*); we—wych elm (*Ulmus glabra*); ca—common ash (*Fraxinus excelsior*); Ib—Iberian white birch (*Betula celtiberica*); sb—silver birch (*Betula pendula*); bl—black locust (*Robinia pseudoacacia*); ww—white willow (*Salix alba*); sm—sugar maple (*Acer saccharum*). ^a^ Types of extractions: SFE—supercritical fluid ex.; UAE—ultrasound-assisted ex.; CE—conventional ex.; MAE—microwave-assisted ex. ^b^ TPC—total phenolic content, expressed as mg gallic acid equivalent per g dry weight of bark (mg GAE/g DWB); n/a— not available. ^c–f^ Assignment of MAE power for TPC value ranges; rt—room temperature. ^g^ In order from top to bottom: whole bark from the continental (cont.) zone, outer bark from the cont. zone, whole bark from the coastal (coa.) zone, outer bark from the coa. zone. ^h^ As mg GAE/g (not specified what material a gram is), incomparable. ^k^ As mg/g DW (not as GAE, obtained by UPLC-PDA-Q/TOF-MS), incomparable.

### 4.6. Evaluation of Tropical and Subtropical Tree Bark Extracts (Table 6)

The highest TPC value (373 ± 4.2 mg GAE/g DWB) was obtained for goran (*Ceriops decandra*). Most of the presented studies cannot be expressed as mg GAE per dry weight of bark and, due to this, cannot be compared to each other. Slight differences were observed between the TPCs of extracts with different fruit colours [60] and human effects on plant growth [54], but more research is needed. Phytochemical screening was the dominant qualitative method for studies in this table, and only groups of compounds were identified.

**Table 6:** Phytochemical extraction from bark of tropical and subtropical trees *—comparison of selected recent studies (since 2018).

Ref.	Solvent Type, Temperature of Extraction	Extraction Method ^a^, Time	TPC (mg GAE /g DWB) ^b^	Extraction Yield (% DW)	Identified Abundant Compounds ^c^
[59] ma	ethanol, ~78 °C	SoxE (3 times refluxed)	60.25	n/a	tannins, flavonoids, carbohydrates, steroids
	ethyl acetate, ~77 °C		63.00	n/a	tannins, flavonoids
	chloroform, ~61 °C		36.25	n/a	tannins, flavonoids, steroids
	petroleum ether, 42–62 °C		29.75	n/a	tannins, flavonoids
[60] wa	methanol	ME	0.34 (pink) 0.34 (red) ^d^	7.58 (pink) 6.48 (red) ^d^	phenols, flavonoids, saponins, triterpenoids, alkaloids
	ethyl acetate		0.26 (pink) 0.30 (red) ^d^	2.90 (pink) 2.44 (red) ^d^	
	n-hexane		0.17 (pink) 0.21 (red) ^d^	0.52 (pink) 0.54 (red) ^d^	
[52] em	methanol, rt	ME	287.16 ± 2.14 ^f^	n/a	phenols, steroids, alkaloids
	methanol– ethyl acetate ^e^		362.88 ± 1.89 ^f^		
	methanol– n-hexane ^e^		15.47 ± 0.38 ^f^		
[64] re	methanol	ME	366.43 ± 11.52 ^f^	4.24	alkaloids, steroids, tannins, xanthones, reducing sugars
[53] ka	n-hexane	ME	187.37 ± 0.06 ^g^	n/a	xanthones
	dichloromethane		127.84 ± 0.05 ^g^		
	ethyl acetate		116.65 ± 0.06 ^g^		
	methanol		73.40 ± 0.11 ^g^		
[65] kt	ethanol	UAE	451.07 ± 3.35 ^g^	n/a	n/a
[65] ct	ethanol	UAE	327.60 ± 2.79 ^g^	n/a	n/a
[65] jf	ethanol	UAE	90.33 ± 0.23 ^g^	n/a	n/a
[74] go	water, 80 °C	CE, 3 h	373 ± 4.2	n/a	n/a
[54] za	methanol	SoxE, 3 days	185.15 ± 1.22 ^g^ (wild) 171.13 ± 6.73 ^g^ (cultivated)	n/a	n/a

* Compared tropical and subtropical trees (abbreviations in Ref. section): ma—mangrove apple (*Sonneratia caseolaris*); wa—water apple (*Syzygium aqueum*); em—*Elaeocarpus mastersii* King; re—resin tree (*Dipterocarpus alatus*); ka—kandis (*Garcinia forbesii*); kt—kusum tree (*Schleicera oleosa*); ct—cashew tree (*Anacardium occidentale*); jf—jackfruit (*Artocarpus heterophyllus*); go—goran (*Ceriops decandra*); za—*Zanthoxylum armatum.* ^a^ Types of extractions: UAE—ultrasound-assisted ex.; SoxE—Soxhlet ex.; ME—ex. by maceration; CE—conventional ex. ^b^ TPC—total phenolic content, expressed as mg gallic acid equivalent per g dry weight of bark (mg GAE/g DWB); n/a—not available. ^c^ Groups of compounds (phytochemical screening). ^d^ Colour of fruits of water apple; rt—room temperature. ^e^ Concentrated methanol extract was partitioned using n-hexane and ethyl acetate. ^f^ As mg GAE/g DW (not specified DW—extract/bark), incomparable. ^g^ Comparable only with others in the same study, expressed as mg GAE per dry weight of extract (cannot be converted to mg GAE per dry weight of bark without extraction yield).

### 4.7. Summary of Survey

Although the identification of phytochemicals was limited to the standards selected by the researchers, some trends could be observed. Polyphenols and their derivatives were the most abundant type of extractives for all groups of trees. Other common compounds were guaiacol and their derivatives, stilbenes and their derivatives, and simple phenolic acids. All the above-mentioned compounds are aromatic, but cycloaliphatic extractives (quinic acid, dehydroabietic acid) and aliphatic extractives (palmitic acid) are also present. The majority of the extractives have at least two hydroxyl groups, which makes them interesting in terms of polymer synthesis. This means that bark extracts, as a relatively uniform mixture, can be a source of substances with similar properties, which have potential as commercial substrates for the polymer industry.

## 5. Application of Bark Extracts in Thermosetting Polymers

### 5.1. Types of Thermosetting Polymers

Thermosetting polymers, also called resins, are a group of synthetic materials that include polyurethanes (PUR), epoxy and polyester resins. Also, formaldehyde-based resins, such as phenol–formaldehyde (PF), urea–formaldehyde (UF) and melamine–urea–formaldehyde (MUF), constitute a group classified as thermosets. Other polymer materials that have a dense, crosslinked structure, a high molar mass and good mechanical properties can be called resins.

### 5.2. Formaldehyde-Based Resins and Alternative Adhesives

PF, UF and MUF resins, which are popular adhesives for plywood and veneer, are rather obvious types of thermosets wherein bark phytochemicals, especially polyphenols and tannins, can be used. The main drawback of formaldehyde-based resins is formaldehyde emission, which can be reduced by modifications or removed for formaldehyde-free alternatives (e.g., amine-based). Bark phytochemicals can be useful in both solutions.

Hajriani et al. paid attention to the importance of the optimal formulation of tannin from the bark of Merkus pine (*Pinus merkusii*), formaldehyde and resorcinol mixtures for PF adhesives [69]. They observed a correlation between the tannin structure with the presence of resorcinol and a relatively low (approx. 5%) formaldehyde demand, which should result in low emissions. Hendrik et al. proposed a similar formaldehyde–resorcinol-based PF adhesive with tannin bark extract from a fast-growing tree—mangium (*Acacia mangium*). The obtained resin partially meets the requirements of Japanese standards (JAS 234, in terms of moduli of elasticity and rupture) and also shows very low formaldehyde emission [68]. Commercial PF was copolymerised with tannins from black wattle (*Acacia mearnsii*) bark in different ratios (20%, 30% and 40% of PF resin solids), and the obtained composites were characterised by better thermal stability and faster curing with lower shear strength [75].

The UF resin was modified with small amounts (2.5% and 5%) of bark extracts of maritime pine (*Pinus pinaster*) and wych elm (*Ulmus glabra*) to obtain phenol–urea–formaldehyde (PUF) resins [70]. PUF adhesive with 2.5% maritime pine extract had improved bonding shear strength (more than 20% higher) for pine plywood. In addition, reduced formaldehyde emission was observed for all compositions with wych elm bark extract (from 1 mg to 5.5 mg per 100 g of oven-dried panel) and maritime pine extract (from 3 mg to 5 mg per 100 g of oven-dried panel). Tannins extracted from the bark of goran (*Ceriops Decandra*) were also added to commercial UF resin and compared to commercial UF and a tannin-only adhesive [74]. The PUF obtained with 25% tannin content was optimal in terms of water resistance and mechanical and adhesive properties. Also, the addition (10%) of finely ground beech bark as a filler for a UF adhesive for plywood provided lower formaldehyde emission and better mechanical properties, which are the result of the presence of bark extractives in the UF adhesive [76]. However, the higher content of filler (15–20%) was too viscous to prepare with laboratory equipment.

Janceva et al. synthesised adhesives from bark extracts (with amounts of condensed tannins (CTs)) of grey alder (*Alnus incana*) and black alder (*Alnus glutinosa*) combined with polyethyleneimine (CTs-PEI) [77]. CTs-PEI was also mixed with ultra-low-emission PF resin (ULEFR). These mixed adhesives (40–60% CTs-PEI substitution) show comparable mechanical properties (modulus of elasticity, shear strength) to clear ULEFR. In the case of CTs-PF resin, formaldehyde emissions were two times lower compared to those of conventional PF adhesives. Another formaldehyde-free alternative adhesive based on Monterey pine (*Pinus radiata*) bark extract and hexamethylenetetramine (7%) with a low solid content (30%) had properties similar to those of commercial PF resin [78]. Garcia et al. conducted a systematic study of polyphenolic resins synthesised with commercial CTs of maritime pine bark (*P. pinaster*) and eleven different aldehydes as formaldehyde alternatives [79]. They observed an increase in the T_g_ and bulk density of resins as a function of the aldehyde chain length. The type of aldehyde chain (aliphatic or aromatic, functionality, length) may also be useful for tailoring the properties of resins (e.g., lower ratio of carbon and hydrogen to other elements improves fire resistance). All collected data are summarised in Table 7.

**Table 7:** Formaldehyde-based resins and alternative adhesives—summary.

Polymer Matrix	Extract Source	Extract Content	Properties	Ref.	Comments
PF	merkus pine bark	over 50% (not specified)	liquid adhesive (solid content from 14% to 17%) with Stiasny number from 50% to 83% and low formaldehyde demand (5%)	[69]	only formulations of tannins with resorcinol and formaldehyde were tested, no mechanical tests of adhesive
PF	mangium bark	ca. 80%	laminate with mangium wood partially meets the JAS 234 standard—slightly lower moduli of elasticity and rupture and good level of formaldehyde emission (very low)	[68]	-
PF	black wattle bark	20%, 30% and 40%	copolymers with better thermal stability, faster curing and lower shear strength than commercial PF	[75]	-
PF	maritime pine bark	40%, 50%, 67%	Tg and bulk density correlation with aldehyde chain length and functionality and unsaturation	[78]	PF
PUF	maritime pine bark	2.5% and 5%	improved bonding shear strength (more than 20% higher), reduced formaldehyde emission	[70]	tested as adhesive for pine plywood
PUF	wych elm bark	2.5% and 5%	reduced formaldehyde emission	[70]	tested as adhesive for pine plywood
PUF	goran bark	25%	reduced moisture content, comparable water absorption and better mechanical properties than commercial UF	[74]	all properties for particleboards; mechanical tests: tensile strength, moduli of elasticity and rupture
UF	beech bark (passive extraction)	10%	reduced formaldehyde emission, equal or higher mechanical properties (modulus of rupture, thickness swelling, bonding quality) compared to plywood with commercial UF	[76]	tested as adhesive for beech plywood
ULEFR	grey alder and black alder bark	40–60%	comparable mechanical properties (modulus of elasticity, shear strength) to clear ULEFR, reduced formaldehyde emission	[77]	tested as adhesive for birch plywood and pine wood particleboards
amine-based PF alternative	Monterey pine bark	90–95%	liquid adhesive (solid content—30%) with properties similar to those of commercial PF resins, reduced formaldehyde emission)	[78]	-

### 5.3. Polyurethanes

Polyphenols, tannins and lignin [80] are potential substrates for the synthesis of PUR as polyol alternatives due to the presence of hydroxyl groups, which react with diisocyanates or their less toxic derivatives (NIPU—non-isocyanate polyurethane).

PUR based on tannins extracted from mangium (*Acacia mangium*) bark and polymeric diphenylmethane diisocyanate (pMDI) was used in the impregnation process of ramie fibres, enhancing their thermal and mechanical properties [81]. Mangium tannin-based PUR for ramie fibre impregnation was compared with the isocyanate-free version of PUR from this extract (NIPU). NIPU was prepared in a reaction with dimethyl carbonate and hexamethylenediamine instead of pMDI [82,83]. Both types of bio-based PUR improved the mechanical parameters of the fibres (isocyanate-based—higher modulus of elasticity; isocyanate-free—higher tensile strength). PUR based on pMDI provided better thermal properties. The most recent study focused on the optimisation of mangium tannin-based NIPU [83].

Higher degradation temperatures were also observed for PUR foam with added hydroxybutylated tannin from Monterey pine (*Pinus radiata*) bark [84]. Hydroxyalkylation was selected for the modification of tannins, which improved their viscosity to a level similar to that of commercial polyols in PUR foams. However, the addition of these homoalkylated tannins still resulted in foam brittleness and a higher density. D’Souza et al. observed similar characteristics (higher degradation temperatures and density, brittleness) of foams made by adding (about 13%) liquefied lodgepole pine (*Pinus contorta*) bark infested with mountain pine beetle [85]. Liquefaction was conducted at two temperatures (90 °C and 130 °C), and different product profiles were obtained. Also, the produced PUR foams had different properties. The 90 °C bark-based foam contained secondary hydroxyls (from sugars) and a foaming behaviour more similar to that of polypropylene glycolfoam based on glycerol (PPG-G). On the other hand, a large amount of low-molecular-weight compounds was disadvantageous to foam properties. The 130 °C bark-based foam (high-molecular-weight, well-functionalised compounds) had a lower density and better elastic properties (elastic modulus and compression strength).

Phytochemicals can also be used as natural photostabilisers for PUR-based coatings. Bark extracts from Chinese fir (*Cunninghamia lanceolata*) were added to a UV-curable waterborne polyurethane-acrylate (PUA) coating, and a relationship between higher TPC and better photostabilisation efficiency was observed, especially for the ethanolic extract [72]. For mimosa (*Accacia mollissima*) bark extract added to PUR varnish, a decrease in surface glossiness was observed, with the exception of the mixture with 10% bark extract, which provides better impregnation and increased glossiness than the tested commercial impregnating agent [86]. Bio-based aromatic diisocyanates were also obtained from guaiacol and vanillyl alcohol, both available in bark extracts [87]. All collected data about polyurethanes are summarised in Table 8.

**Table 8:** Polyurethanes—summary.

Polymer Matrix	Extract Source	Extract Content	Properties	Ref.	Comments
PUA	Chinese fir bark	2%	coating: increased photostability	[72]	better for higher TPC
PUR	mimosa bark	10%	coating: better impregnation and glossiness	[86]	other extract contents were suboptimal
PUR	Monterey pine bark	25%, 50%, 75%, 100%	foam: improved thermal stability, increased strength and brittleness, deformed cellular structure *	[84]	* only for higher loadings
PUR	lodgepole pine bark	ca 13%	foam: higher degradation temperature and density, increased brittleness	[85]	-
PUR	mangium bark	non-specified	improved thermal stability and crystallinity	[81]	used for impregnation of ramie fibres, different times of impregnation
PUR	mangium bark	non-specified	improved thermal stability, tensile strength and elastic modulus	[82]	used for impregnation of ramie fibres
NIPU	mangium bark	30.7% and 44.4%		[82]	
NIPU	mangium bark	30.7%	as above, optimisation	[83]	used for impregnation of ramie fibres

### 5.4. Polyester Resins

The presence of hydroxyl and carboxylic groups in phytochemicals can be attractive for the synthesis of polyesters. Tannins and other naturally occurring biopolyesters can be applied without other synthetic substrates.

Han et al. selected salicylic acid (present in willow (*Salix* sp.) bark) as a poly(salicylate) homopolymer substrate with a narrow molecular weight distribution and a high glass transition temperature (T_g_) [88]. They obtained a six-membered cyclic *o*-carboxyanhydride of salicylic acid as the intermediate product, which was polymerised via ring-opening polymerisation. Lang et al. proposed a biopolyester adhesive based on torrefied bark of silver birch (*Betula pendula*) as an alternative to hot-melt glue or a tackifying agent [89]. Birch bark is also a source of betulin, which reacts with aliphatic acid dichlorides (from 5 to 8 carbons in the methylene chain) and gives a biopolyester with a T_g_ comparable to that of polycarbonates (150–165 °C) [90]. Also, thermal treatment of such biopolyesters (2 h at 250 °C) provides better chemical resistance to tetrahydrofuran (THF), CHCl_3_ and N-methylpyrrolidone. Moreover, the polycondensation product of betulin and sebacoyl dichloride is a transparent biopolyester. Furthermore, suberin fatty acid hydrolysates (from the outer bark of silver birch) crosslinked with maleic anhydride and octadecyltrichlorosilane can constitute competitive biopolyester coatings for the wood industry, providing improved hydrophobicity and stable anti-ageing properties [91]. Gosecki et al. also proposed a vitrimer based on birch suberin monomers and commercial polyols in the presence of a tin catalyst [92]. The obtained recyclable polymers show good hydrolytic stability and creep resistance. Cho et al. isolated depolymerised suberin derivatives from the powdered bark of cork oak (*Quercus suber*) and obtained syntactic biopolyester foams by polycondensation with glycerol (5.77 wt%) [93]. These foams were similar to cork in terms of density and porosity but had a different chemical structure (higher content of suberin), which caused different viscoelastic properties (higher tan δ and a more viscous character).

Poly(δ-decalactone) and poly(δ-dodecalactone), two other types of polyesters, can be synthesised via the ring-opening polymerisation of δ-decalactone and δ-dodecalactone, respectively [94]. Both lactones were reported as oil ingredients harvested from the bark of the Massoia tree (*Cryptocarya massoia*) [95]. Garcia et al. extracted polyflavonoids from Monterey pine (*Pinus radiata*) bark and proposed their synthesis with poly(lactic acid) (PLA) [96]. The obtained polyester blends were compared to their modified versions, where hydroxypropylated (propylene oxide was used) polyflavonoids were synthesised with PLA instead of raw ones. In addition, blends with poly(ethylene glycol) (PEG) were obtained. All types of blends induced the crystallisation of PLA, but the PLA–polyflavonoid–PEG blends also had reduced transitional temperatures because of the breakage of the intermolecular hydrogen bonds and increased chain mobility. Furthermore, the bark tannins of Monterey pine (*Pinus radiata*) were hydroxypropylated (also with propylene oxide) by Bridson et al. [97]. Hydroxypropylated tannins were blended with PLA using the melt-spinning method. Surprisingly, a more amorphous character was observed for blends with hydroxypropylated bark tannins (as opposed to the conclusions of Garcia et al. [96]). These differences may be related to the type of selected PLA, the extraction parameters and the blending procedure. This suggests that the impact of polyphenols on PLA blends is more complex and needs further investigation. All collected data are summarised in Table 9.

**Table 9:** Polyester resins—summary.

Polymer Matrix	Extract Source	Extract Content	Properties	Ref.	Comments
polyester	willow bark	almost 100%	high T_g_, rapid polymerisation with a narrow distribution of molecular weights	[88]	-
polyester	torrefied bark of silver birch	100%	high adhesion—alternative to hot-melt glue or tackifying agent	[89]	-
polyester	silver birch bark	52%	T_g_ at 150–165 °C, good chemical resistance	[90]	made from unsaturated betulin with sebacoyl chloride and pyridine
polyester	silver birch bark	70%	improved hydrophobicity and stable anti-ageing properties	[91]	made from suberin fatty acids with maleic anhydride
polyester	silver birch bark	non-specified	good hydrolytic stability and creep resistance	[92]	made from suberin with polyol and tin catalyst
polyester	cork oak bark	non-specified	foams similar to cork	[93]	made from depolymerised suberin with glycerol and bismuth catalyst
poly(lactic acid) blend	Monterey pine bark	10%, 20%, 30%, 40%	improved processability of PLA, more crystalline character	[96]	also hydroxypropylated extract was tested
poly(lactic acid) blend	Monterey pine bark	25%	amorphous character	[97]	hydroxypropylated extract, obtained by melt-spinning

### 5.5. Epoxy Resins

The hydroxyl groups of phytochemicals can be epoxidised with chlorohydrin to obtain epoxy compounds for resin synthesis [98] or react with an epoxy ring to form epoxy resins (in the presence of a specific catalyst). Also, the reaction between carboxyl groups and epoxy rings is a common synthesis method of epoxy resins. 

Bridson et al. [99] confirmed the reactivity of the Monterey pine (*Pinus radiata*) bark tannin with simple aliphatic epoxides (propylene oxide, butylene oxide and hexylene oxide) in the presence of triethylamine (hydroxylation). The hydroxylated products had reduced viscosity compared to natural tannins, but viscosity and T_g_ increased with the increasing aliphatic chain length of the epoxide. Extractives (water-soluble fraction and fraction insoluble in water) from Monterey pine (*Pinus radiata*) bark were added to commercial epoxy-polyamide resin as copolymers [100]. Both fractions are competitive as corrosion inhibitors (the water-soluble fraction performs better). This effect is probably the result of the formation of complexes between Fe^3+^ ions and adjacent hydroxyl groups of the B-ring of polyphenols. Epoxidised extracts from the bark of various trees were reported recently. Shnawa prepared tannin-based epoxy resin from eucalyptus (*Eucalyptus* sp.) bark and compared it with a commercial product [101]. The crosslinking process with different ratios of the amine as a curing agent was more sluggish for tannin-based epoxy as a result of the steric effects of the tannin structure, according to the author. Despite this, the commercial resin also had reduced viscosity (presence of 1,6-hexanediol diglycidyl ether, 10–30%), which could also improve reactivity. Only blends of commercial epoxy with up to 20% tannin-based epoxy maintained curing properties similar to those of neat commercial epoxy. Bog-myrtle (*Myrica gale*) bark extract was another mixture of tannins functionalised with epoxy groups. The obtained epoxy was copolymerised with chitosan and applied as a paper coating that provides resistance to oil and water [102].

Another solution was the addition of the aforementioned epoxy to a commercial one as a strengthening agent (5%) [103]. It was successful: a significant improvement in flexural, tensile and impact strengths was obtained with the maintenance of chemical and thermal resistance. Chen et al. epoxidised eucommia ulmoides gum sourced from *Eucommia ulmoides* bark and added it to the epoxy coating as a nanofiller [104]. Composites with up to 1% of such filler had improved tensile strength and enhanced corrosion performance as a result of a higher crosslink density.

An interesting type of compound is magnolol extracted from houpu magnolia (*Magnolia officinalis*) bark, which can be turned into epoxy by the epoxidation of allyl groups (Prilezhaev reaction with 3-chloroperoxybenzoic acid in dichloromethane solution, without catalyst) instead of hydroxyl groups [105]. Unreacted hydroxyl groups and created epoxy groups reveal self-curing behaviour above 120 °C. The self-curing activation energy was relatively low (94.8 kJ/mol) compared to the activation energy (77.0 kJ/mol) of the curing reaction of this epoxy with an amine (4,4’-methylenedianiline), which obviously acts as a catalyst. Furthermore, self-cured magnolol-based epoxy resin had lower flammability compared to other tested systems. In addition, both magnolol-based epoxy resins had antibacterial properties. The commercial availability of magnolol and the unique properties of magnolol-based epoxy have resulted in new research articles on the topic. Cao et al. tested ester functional groups (instead of hydroxyl groups) for magnolol-based epoxy as an alternative [106]. They observed the necessity of an amine hardener as a catalyst for self-curing (only 0.5% or 1%), but the obtained thermosets had higher stiffness and toughness compared to commercial epoxy resins based on the diglycidyl ester of bisphenol A (DGEBA). Zhang et al. proposed magnolol-based epoxy as a reinforcement for bio-based aerogel made of a chitosan skeleton [107]. Wei et al. synthesised a reactive flame-retardant—diglycidyl ether of magnolol phosphine oxide—and added it to commercial epoxy [108]. Epoxy systems with 10% and 15% flame-retardant had significantly lower heat production and slightly increased smoke production in the cone calorimeter test. Also, the limited oxygen index in the UL-94 test was measured (37.0% and 41.5%, respectively, both V-0 rating). All data collected on epoxies are summarised in Table 10.

**Table 10:** Epoxy resins—summary.

Polymer Matrix	Extract Source	Extract Content	Properties	Ref.	Comments
epoxy	Monterey pine bark	22%, 28%, 33%, 50%	oligomers with various viscosities and T_g_, affected by chain length	[99]	reactivity tests
epoxy	Monterey pine bark	non-specified	competitive as corrosion inhibitor	[100]	-
epoxy	eucalyptus bark	20%, 40%, 60%	slightly faster curing for 20% content	[101]	bark extract was epoxidised
epoxy	bog-myrtle bark	non-specified	oil and water resistance	[102]	as paper coating
epoxy	bog-myrtle bark	5%	significant improvement in flexural, tensile and impact strengths	[103]	as strengthening agent
epoxy	eucommia ulmoides bark	1%	improved tensile strength and enhanced corrosion performance	[104]	as nanofiller
epoxy	houpu magnolia bark	over 90%	lower flammability and antibacterial properties	[105]	self-curing ability
epoxy	houpu magnolia bark	over 90%	higher stiffness and toughness compared to DGEBA	[106]	added an amine catalyst for self-curing
epoxy-chitosan copolymer	houpu magnolia bark	9%, 16.6%, 23.1%, 28.6%, 33.3%	improved flame retardancy	[107]	as hard segments for chitosan-based aerogel
epoxy	houpu magnolia bark	10%, 15%	significantly lower heat production and slightly increased smoke production, V-0 rating for UL-94 test	[108]	as a reactive flame-retardant

### 5.6. Other Thermosets

Magnolol can also be a precursor for other thermosets. Magnolol and honokiol functionalised with 4-nitrophthalonitrile (hydroxyl group substitution) are able to form phthalonitrile thermosets via self-curing reactions [109]. These resins exhibit high thermal stability (T_g_ > 500 °C) compared to similar resins obtained from petroleum-based precursors. The allyl groups of magnolol are also useful for thiol-ene click chemistry. Adhesive synthesised from magnolol and a tetrafunctional thiol—pentaerythritol tetra-(3-mercaptopropionate)—can be used to bond different types of materials (PVC, steel, wood, glass), even in a wet environment (adhesion strength greater than 7.5 MPa and 6.0 MPa after immersion in hot water for 3 h) [110]. Furthermore, an antibacterial effect was also observed. Weems et al. tested five different terpenes with a multifunctional thiol to obtain thermosetting resins via thiol-ene photopolymerisation [111]. They obtained promising materials for 3D printing with mechanical properties more similar to those of elastomers and thermoplastics. Recent studies proposed the biomimetic crosslinking of silicone elastomers by polyphenols with an allyl group (i.e., eugenol) in the presence of a platinum catalyst [112], which can also be attractive for the synthesis of thermoset–silicone copolymers. Also, combustion behaviour studies of cork and phloem from the bark of different trees [113] suggest that bark after extraction, without relatively flammable extractives, can be an interesting ecological alternative for flame-retardant fillers. The data collected showing the application of phytochemicals in other resins are summarised in Table 11.

**Table 11:** Other resins—summary.

Polymer Matrix	Extract Source	Extract Content	Properties	Ref.
phthalonitrile resin	houpu magnolia bark	42.80%	high thermal stability (T_g_ > 500 °C)	[109]
thiol-ene resin	houpu magnolia bark	35.3%, 45.0%, 52.2%, 59.2%, 68.6%	good adhesive in a wet environment, antibacterial properties	[110]
thiol-ene resin	limonene	36.2%	3D printing material with mechanical properties similar to those of elastomers and thermoplastics	[111]

## 6. Discussion and Perspectives

**Figure 4:**
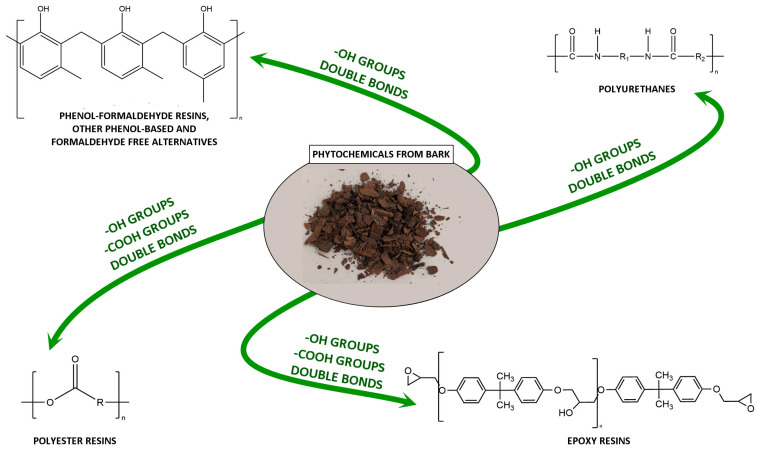
Functional groups of bark phytochemicals and their applicability in the synthesis of thermosetting polymers.

The evaluation of data collected for six groups of trees revealed an interesting relationship between the extraction time and the type of solvent for some types of bark trees. Relatively short extraction times and a temperature close to the boiling point of the solvent are favourable, although each type of bark has unique and distinctive extraction needs. However, as stated by Warlo et al. [114], bark extraction should be designed for the stage of existing industrial processes for a better use of resources. The description of phytochemicals present in the extractives of different trees in this article, as well as their structures and TPC values, is an introduction to the discussion of the attractiveness of the most popular bark in terms of possibilities arising from extractable phytochemicals. Polyphenols, tannins and alcohols need complex investigation as carriers of reactive hydroxyl groups. The location of hydroxyl groups is also an important factor. Pinnataip [115] and Janceva [77] indicate that the catechol structure present in most bark compounds (only reported in this article: astringin, flavan-3-ols, taxifolin, myricetin, caffeic acid, quercetin, procyanidin, gallic acid, protocatechuic acid and selected glycosides of the listed compounds) is sensitive to oxidation and therefore reactive in the form of orthoquinone with primary and secondary amine groups. It is an important attribute for the chemistry of epoxy resins and amine-based formaldehyde-free alternatives of PF resins (Figure 4).

Also, functional groups with double bonds (vinyl, allyl, etc.) discussed in the “Other Thermosets” Section [110,111] may be interesting for the creation of a polymer network. Most phytochemicals found in bark do not have multiple easily accessible double bonds, but a relatively large group has at least one such double bond (only reported here: stilbenoids and stilbene glycosides, caffeic acid, 4-vinyl guaiacol, syringin, triterpenoids). This makes it possible to use thiol-ene chemistry as a mechanism, which may support hydroxyl-based curing reactions. Also, the Prilezhaev reaction [105] is an interesting approach to the epoxidation of groups with double bonds, the advantage of which is the availability of hydroxyl groups along with epoxy groups in one compound (enabling homopolymerisation similar to magnolol). Phytochemicals are mainly aromatic or cycloaliphatic structures and provide greater stiffness of the polymer chain and better thermal properties but may be less reactive because of steric hindrance. Furthermore, the more complex architecture of compounds such as condensed tannins (CTs) must also be taken into account. As oligomeric structures, they are convenient as a resin backbone, but the main drawback of their application is alkaline degradation resulting from the opening of the C_15_ flavonoid unit [79].

## 7. Conclusions

All of the advantages and disadvantages mentioned above should be helpful in selecting the most suitable compounds for each specific application. However, selection usually requires separation processes, because compounds are generally components of the mixture. This approach should be considered as long as the separation is economically justified. The summary of the presented polymer solutions with phytochemicals reveals two main application methods. The first method—applying them as additives to commercial thermosets—may be convergent with a selective approach if the addition of phytochemical provides sufficient benefits. However, a small amount of phytochemicals does not change a petroleum-based resin to a bio-based one (even if marketing specialists say so). It is still only a palliative measure for the issues mentioned in the “Introduction” Section. The second method, the bio-based thermoset (or the main part of it), as an alternative to the commercial one, should provide a better solution for managing bio-waste and replacing fossil-based chemicals. Unfortunately, a selective approach may result in higher production costs for such alternatives and lower competitiveness than existing solutions (only significantly better properties will justify the “bio-choice”). To avoid expensive separation stages, a more holistic approach is needed. The selection of phytochemicals should be less rigorous and more focused on groups of compounds having common characteristics that fit the desired application. TPC can be a useful standardisation tool for such extracts as a relatively fast and simple method (but a unified expression of the TPC values should be used, as stated in Section 4). Exclusion should be taken into account only for compounds that significantly worsen the properties of the final product. However, the question about the competitiveness of such bio-resins compared to commercial solutions remains. Of course, none of these approaches and application methods is the most favourable. Each has advantages and disadvantages that must be considered when designing new thermoset materials.

## Data Availability

The data presented in this study are mostly openly available. DOI addresses are provided in the References Section.
